# Peripheral Neuropathy in Systemic Autoimmune Rheumatic Diseases—Diagnosis and Treatment

**DOI:** 10.3390/ph16040587

**Published:** 2023-04-14

**Authors:** Jean Marcos De Souza, Thiago Junqueira Trevisan, Samara Rosa Sepresse, Ana Carolina Londe, Marcondes Cavalcante França Júnior, Simone Appenzeller

**Affiliations:** 1 Department of Internal Medicine, School of Medical Science, University of Campinas, Campinas 13083881, Brazil; souzajm@unicamp.br; 2 Department of Orthopedics, Rheumatology and Traumatology, School of Medical Science, University of Campinas, Campinas 13084971, Brazil; thiagojt@unicamp.br; 3 Autoimmunity Laboratory, School of Medical Science, University of Campinas, Campinas 13083881, Brazil; s264429@dac.unicamp.br (S.R.S.); alonde@unicamp.br (A.C.L.); 4 Graduate Program in Child and Adolescent Health, School of Medical Science, University of Campinas, Campinas 13083881, Brazil; 5 Post-Graduate Program in Physiopathology, School of Medical Science, University of Campinas, Campinas 13083881, Brazil; 6 Department of Neurology, School of Medical Science, University of Campinas, Campinas 13083888, Brazil; mcfjr@unicamp.br

**Keywords:** rheumatic diseases, connective tissue diseases, systemic vasculitis, peripheral neuropathy

## Abstract

Peripheral neuropathy (PN) is frequently observed in systemic rheumatic diseases and is a challenge in clinical practice. We aimed to review the evidence on the subject and proposed a comprehensive approach to these patients, facilitating diagnosis and management. We searched the MEDLINE database for the terms (and its respective Medical Subject Headings (MeSH) terms): “peripheral neuropathy” AND “rheumatic diseases” OR “systemic lupus erythematosus”, “rheumatoid arthritis”, “Sjogren syndrome”, and “vasculitis” from 2000 to 2023. This literature review focuses on the diagnostic workup of PNs related to systemic lupus erythematosus, Sjögren’s syndrome, rheumatoid arthritis, and systemic vasculitis. For every type of PN, we provide a pragmatic flowchart for diagnosis and also describe evidence-based strategies of treatment.

## 1. Introduction

Rheumatic diseases represent a vast group of immune-mediated inflammatory conditions including autoimmune connective tissue diseases and systemic vasculitides. Neurologic manifestations are a hallmark of many of these entities due to many types of inflammatory attack on neuronal tissue, and also due to nerve entrapment, drug toxicity, and metabolic vasculopathy. Because the pathogenic mechanisms of inflammatory neuropathies are still somewhat obscure and many of them are at least partially reversible, great attention is usually paid to recognizing the immune-mediated neuropathies in the rheumatic patient.

One of the most common neurologic manifestations of the immune-mediated rheumatic diseases is peripheral neuropathy (PN) [1]. It is especially recognized in Sjögren syndrome (SS), occurring in up to 60% of patients [2], systemic lupus erythematosus (SLE), in up to 15% [3,4], and systemic vasculitis, in up to 70% [5]. The patterns of clinical presentation are diverse, with different neurological syndromes occurring in the same disease, making it very challenging for most practitioners not only to attribute the PN to inflammation itself but also to determine the specific topography of the insult. Because some treatments may differ depending on the type of PN [6], it is of utmost interest for the clinician to be able to understand as best as possible the clinical picture.

This literature review focuses on the diagnostic workup of PNs related to SLE, SS, rheumatoid arthritis (RA), and systemic vasculitis. For every type of PN, we also describe strategies of treatment.

## 2. Methodology

We searched the MEDLINE database for the terms (and its respective Medical Subject Headings (MeSH) terms): “peripheral neuropathy” AND “rheumatic diseases” OR “systemic lupus erythematosus”, “rheumatoid arthritis”, “Sjogren syndrome”, and “vasculitis”.

The search strategy was performed on 6 January 2023 and included review articles from 2000 to 2023, published in English, Spanish, or Portuguese. Instead of describing the neuropathies related to each rheumatic disease (something that was already and successfully carried out before) [1], we decided to start from the neurologic syndromes aiming to narrow the diagnosis down to the main rheumatic conditions. Given a specific neurological syndrome, we described the most probable associated rheumatic conditions and a reasonable diagnostic flow.

Furthermore, treatment options are provided for each rheumatic-disease-associated PN.

## 3. Review

Notably, some authors used a classification of neuropathies distinct from the one we used in this review. Especially in SS, some authors used the term “nonataxic sensory neuropathy” for the group comprising sensory polyneuropathy (without ataxia), pure small fiber neuropathy and trigeminal neuropathy [7,8]. Nevertheless, the same entities are covered.

### 3.1. Small Fiber Neuropathy

Small fiber neuropathy (SFN) is characterized by the degeneration of thinly myelinated A-delta fibers and unmyelinated C-fibers, sparing the thicker fibers related to motor functions, vibration, and proprioception [9,10]. Increased awareness has led to an increased frequency of SFN being reported in the past years [11]. In rheumatic disorders, the hypothesized pathogeneses include direct damage to the nerve axons by cytokines [12], antibodies [9], or cytotoxic cells [13].

Patients most often complain of pain with neuropathic features (e.g., burning, itchy, or prickling in quality) in the distal surface of feet. The pain is usually constant but may be worsened by light stimuli, such as the rubbing of clothes in the affected areas. As the disease progresses, the pain tends to ascend proximally towards the knees, and eventually the same process can be seen in the hands ascending to the elbows [10]. The loss of thermal sensation and numbness are also reported [9]. Although most patients present this symmetric phenotype, some patients might experience patchy, asymmetric, and proximal islets of symptoms in the trunk, face, scalp, or tongue [9,14]. In the latter, the topography of injury is presumably the dorsal root ganglia’s thin fibers [1]. Because thin fibers carry most of the visceral autonomic innervation, patients may also present with autonomic dysfunction, expressed by postural hypotension or tachycardia, bowel disturbances, sweating complaints, and sexual dysfunction [10,11].

The physical examination most frequently reveals a patient with pinprick and thermal sensation loss and preserved motor functions, coordination, deep reflexes, and discriminative touch [10].

The confirmation of SFN is challenging because nerve conduction studies are typically normal. “Quantitative sensory testing” (QST) is the broad name for quantitative methods to assess all forms of sensation through parametrized physical examination and measurement tools [10,15]. It can be used for the diagnosis of SFN because it is capable of discriminating the forms of sensation carried by thin and thick fibers. Nevertheless, QST is a psychophysical test and may depend on patient cognition and collaboration to be reliable.

The use of a skin biopsy to count intra-epidermal nerve fiber density is probably the most used and studied method for the diagnosis of SFN [16]. For diagnostic purposes, patients with SFN have a decrease in intra-epidermal nerve fiber density [17]. Techniques involving evoked potentials (EP) can also be used for the evaluation of SFN, with variable availability depending on the scenario. Pain-related EP, for instance, can be obtained by the selective electrical stimulation of superficial layers of the dermis [18]. This method can be used to grasp the activity from A-delta-fibers and has been successfully tested in diabetes-related SFN [19]. Another possibility to isolate thin fibers is laser EP, in which short heat pulses are emitted from a CO_2_ laser on the skin. The delay in EP has been described and standardized, being long for A-delta and even longer for C fibers [9]. The method is probably also useful for the diagnosis of SFN [20] but is not widely available, and its particularities might make C fibers difficult to isolate [9]. Finally, contact heat EP relies on the application of a very precise and fast method of delivering high temperatures of up to 70 °C to the skin [9,21]. The method is safe and standardized values have already been tested [22], but extensive research on the accuracy for SFN is still lacking.

Up to 30% of patients with SFN might have SS [23], making it the most likely rheumatologic disorder to which SFN is related by far. In SLE, approximately 3% of patients will present with SFN [1,3,4]. Although SFN has been described in systemic vasculitis, it is almost always associated with significant nerve damage so is categorized into the groups of more severe polyneuropathies. Finally, most studies with RA describe sensory polyneuropathy as a common feature, but SFN itself is scarcely appreciated [24,25]. This may be due to the difficult diagnostic framework of SFN, so that the real prevalence of it in RA is probably underestimated. Because RA is largely the most prevalent autoimmune rheumatic condition, it will be a frequent cause of SFN when the differentials departing from the neurological picture are considered [26].

**Figure 1:**
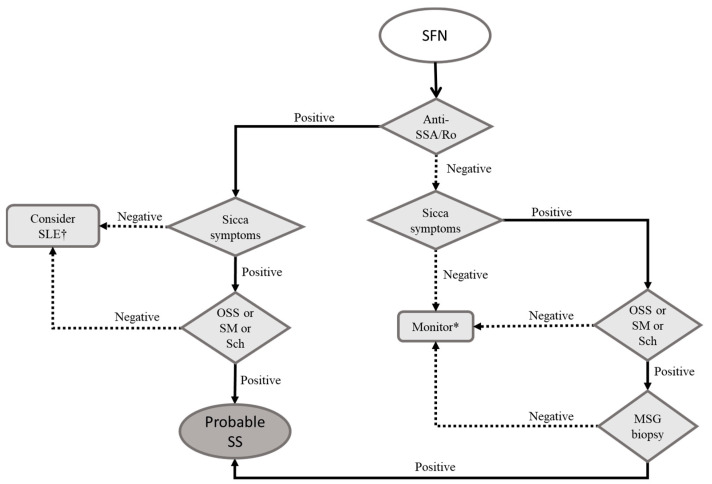
Proposed diagnostic flowchart for patients with small fiber neuropathy. Notes: Anti-SSA/Ro is the main autoantibody related to Sjögren’s syndrome and part of the contemporary diagnostic criteria. Sicca symptoms refer to the complaints related to dryness of the mouth, eyes, skin, and genital and respiratory tracts. Legend: MSG: minor salivary gland; OSS: ocular staining score (ophthalmic exam with specific dyes aiming to document and quantify eye surface’s lesions); Sch: Schirmer’s test (quantification of lacrimal flow volume by time unit); SFN: small fiber neuropathy; SLE: systemic lupus erythematosus; SM: unstimulated sialometry (simple quantification of salivary flow volume by time unit); SS: Sjögren syndrome. * Consider treating neuropathic pain and repeating functional tests after 6 months. † Consider testing for anti-DNA and anti-Sm.

Therefore, we suggest that SS, SLE, and RA are the rheumatic conditions that should be sought in a patient with SFN. Figure 1 highlights a diagnostic flowchart for patients with SFN and suspected autoimmune rheumatic disorder.

### 3.2. Treatment of SFN

The evidence on therapies regarding SFN is limited. However, studies so far regarding inflammatory forms of SFN highlight the unpromising results of immunosuppressive therapies, including glucocorticoids [8], immunoglobulin [8], and rituximab, an anti-CD20 monoclonal antibody related to the inhibition of B-lymphocytes [27]. In practical guidelines, the European Alliance of Associations for Rheumatology (EULAR) suggests only symptom control for the pure sensory neuropathy related to SS [6]. Considering symptomatic management, most guidelines suggest drugs utilized in neuropathic pain, such as gabapentin, pregabalin, tricyclic antidepressants, duloxetine, venlafaxine, topical lidocaine, and/or tramadol [10,28]. To the best of our knowledge, SFN itself has been evaluated only once in a controlled trial, with equal benefits from tramadol or gabapentin in relation to a placebo [29]. If pain control persistently fails or progressive/debilitating symptoms remain, we believe that, based on small case series, a trial of intravenous immunoglobulin (see Table 1 for suggested dosages) is the most promising approach [30].

### 3.3. Axonal Sensory Polyneuropathy (SPN) and Sensorimotor Polyneuropathy (SMPN)

In SPN/SMPN, aggression towards thicker axons, responsible for the transmission of motor functions, vibratory sensation, proprioception and light touch, is observed. By definition, these axonal disorders should lead to electrophysiologic findings of the reduced amplitude of action potentials without primary changes in conduction velocity [31].

SPN/SMPN are described as the most common peripheral neurologic manifestations of immune-mediated rheumatic diseases, occurring in 40% of RA [24,25], 30% of SS [7,32,33], 7% of SLE [3,34], and less than 10% of systemic vasculitis patients [35,36,37]. Some authors suggest that pure SPN could be associated with ganglion cell destruction by direct cytotoxic lymphocytes, representing the same pathogenesis of dorsal root ganglionopathy (ataxic sensory polyneuropathy) [8]. On the other hand, SMPN is a result of neural toxicity from inflammatory cytokines (including IL-1, IL-6, and TNF), nerve damage induced by autoantibodies, infiltration of nerve fibers by inflammatory cells, and/or vasculopathy [38,39,40].

The clinical picture is characterized by symmetric stocking-and-glove-pattern pain and/or dysesthesia, with slow progression towards the thighs and shoulders. Motor symptoms (weakness of fingers extensors and ankle dorsiflexors) emerge after sensory changes, and in the presence of proprioception deficits, are associated with frequent falls [38].

SPN/SMPN are confirmed with a nerve conduction study combined with needle electromyography, identifying a length-dependent decrease in sensory and motor action potentials in multiple nerves of the extremities [32,38,39]. Because secondary demyelination can be observed in immune-mediated rheumatic disease, the amplitude and velocity of action potentials can also be decreased [34,38,39,41,42].

**Figure 2:**
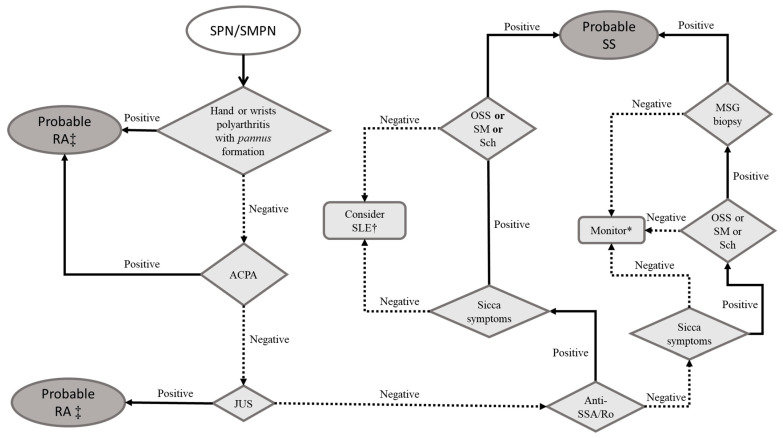
Proposed approach for the patient with pure sensory polyneuropathy (except isolated small fiber neuropathy) or sensorimotor polyneuropathy. Notes: Anti-SSA/Ro is the main autoantibody related to Sjögren’s syndrome and part of the contemporary diagnostic criteria. Sicca symptoms refer to the complaints related to dryness of the mouth, eyes, skin, and genital and respiratory tracts. Legend: ACPA: anti-cyclic citrullinated peptide antibody; JUS: joint ultrasound; MSG: minor salivary gland; OSS: ocular staining score (ophthalmic exam with specific dyes aiming to document and quantify eye surface’s desepithelization); RA: rheumatoid arthritis; Sch: Schirmer’s test (quantification of lacrimal flow volume by time unit); SPN/SMPN: sensory polyneuropathy/sensorimotor polyneuropathy; SLE: systemic lupus erythematosus; SM: unstimulated sialometry (simple quantification of salivary flow volume by time unit); SS: Sjögren’s syndrome. * Treat neuropathy (see text) and either: (1) order anti-Sm and anti-DNA for systemic lupus erythematosus; (2) order antineutrophil cytoplasmic antibody for systemic vasculitides; or (3) reassess in 6 months. † Consider testing for anti-DNA and anti-Sm. ‡ Order rheumatoid factor and anti-cyclic citrullinated peptide antibody (if not carried out yet) for prognostic purposes.

Figure 2 synthetizes our stepwise proposed evaluation in patients presenting with SPN/SMPN suspecting immune-mediated rheumatic diseases.

### 3.4. Treatment of SNP/SMNP

The treatment for SPN and SMPN associated to rheumatic diseases lacks good-quality evidence and is based on small case series and retrospective studies. EULAR’s guideline for Sjögren syndrome suggests intravenous immunoglobulin as the first-line treatment for the motor forms of PN and neuropathic pain treatment (see above, in *small fiber neuropathy* section) for the nonataxic sensory polyneuropathies [6]. Methylprednisolone or cyclophosphamide are considered as second-line treatments [6]. A systematic review for SLE-associated PN reported similar treatments, including glucocorticoids in different dosages [43]. Because they were used frequently with other immunosuppressive drugs, it is hard to grasp their real efficiency. Nevertheless, EULAR recommends “glucocorticoids alone or in combination with immunosuppressive therapy” for SLE-PN. As for intravenous immunoglobulin, the previously mentioned systematic review reported good responses for all the five patients analyzed. No conclusions could be drawn from cyclophosphamide, and rituximab was effective in six out of the eight cases reported [43]. Finally, one retrospective study showed good results with azathioprine [34].

In RA, if there is evidence of disseminated vasculitis (i.e., rheumatoid vasculitis), treatment should consist of glucocorticoid and intravenous cyclophosphamide [44]. If SNP/SMNP is the only manifestation, treatment is extrapolated from the other conditions above.

For systemic vasculitis, particularly for antineutrophil cytoplasmic antibody-associated vasculitis (AAVs), EULAR’s management guidelines do not list PN as a part of the life-threatening situations, suggesting therefore the combination of glucocorticoids (1 mg/kg; maximal 80 mg/kg/day) and methotrexate or mycophenolate mofetil [45].

Our suggested approach involves evaluating patients for severity of the PN. For pure SPN with preserved deep sensation or only minor motor impairment, without an impact on daily living, we suggest the management of neuropathic pain and nonpharmacological strategies, such as physical therapy and acupuncture. These patients should be closely followed for disease progression. For patients with significant motor or deep sensation impairment, we suggest methylprednisolone alone or in combination with cyclophosphamide (Table 1).

### 3.5. Sensory Neuronopathy

This disabling and unusual form of PN is a consequence of the degeneration of the dorsal root ganglia neurons in multiple spinal and cranial levels. It is associated with malignancy, vitamin E deficiency, drugs (e.g., platinum-based chemotherapy), HIV infection, and neurodegenerative conditions [46,47]. When related to rheumatic conditions, SS is by far the most frequently associated disorder [48,49]. In immune-mediated sensory neuronopathies, the pathogenesis is usually well described, with the infiltration of dorsal root ganglia by cytotoxic CD8+ lymphocytes [50]. More recently, some autoantibodies were described in association with SS-related sensory neuronopathy, but their pathogenic role remains unclear [51].

The clinical manifestations include a multifocal and patchy sensory disorder, extending proximally and distally, with all the sensory modalities potentially compromised, often in an asymmetric manner or progression [47,52]. Large sensory fibers are prone to lesion and responsible for the development of proprioceptive ataxia and areflexia. Additionally, small fiber neuropathy occurs, with the neuropathic patterns discussed above. When autonomic nerve fibers are compromised, dysautonomia may be present [48].

The clinical suspicion of a sensory neuronopathy requires NCS and neuroimaging as confirmatory tests [53]. NCS usually shows decreased sensory action potentials’ amplitudes with normal velocity of conduction [48], without a distal predominance in most cases. In addition, magnetic resonance imaging (MRI) of the spinal cord shows hyperintense T2-weighted lesions at posterior columns.

SS should be considered as the first hypothesis in a patient presenting sensory neuronopathy. Because more than 50% of SS patients do not have sicca symptoms at the onset of sensory complaints [47], considering this connective tissue disease in the differential is crucial but not at all instinctive. On the other hand, patients with RA and SLE have been subjects for case reports or single cases in retrospective studies [47,54,55].

**Figure 3:**
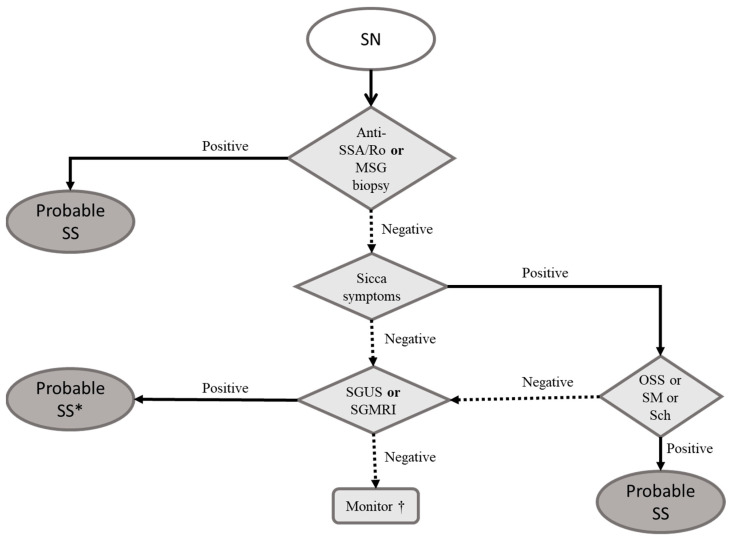
Proposed approach for patients with sensory neuronopathy. Notes: Anti-SSA/Ro is the main autoantibody related to Sjögren’s syndrome and part of the contemporary diagnostic criteria. Sicca symptoms refer to the complaints related to dryness of the mouth, eyes, skin, and genital and respiratory tracts. Legend: MSG: minor salivary gland; OSS: ocular staining score (ophthalmic exam with specific dyes aiming to document and quantify eye surface’s desepithelization); Sch: Schirmer’s test (quantification of lacrimal flow volume by time unit); SGMRI: magnetic resonance imaging of salivary glands; SGUS: ultrasound of salivary glands; SM: unstimulated sialometry (simple quantification of salivary flow volume by time unit); SN: sensory neuronopathy; SS: Sjögren’s syndrome. * Consider new biopsy of minor salivary gland, because the first one might have missed the diagnosis. † Treat aggressively (see text) and consider the unusual diagnosis of systemic lupus erythematosus or rheumatoid arthritis-related sensory neuronopathy. Might order anti-cyclic citrullinated peptide antibody, anti-DNA, and anti-Sm. If negative, consider a new assessment for Sjögren’s syndrome in 6 months.

Thus, we suggest that SS should be sought in all patients without an obvious cause for sensory neuronopathy (e.g., paraneoplastic, viral, or drug-related), regardless of other ocular, articular, or mucocutaneous symptoms [56] (Figure 3).

### 3.6. Treatment of Sensory Neuronopathy

Sensory neuronopathies can be very disabling and early and aggressive treatment should be administered because of a narrow therapeutic window existing for a good response before irreversible damage to ganglia occurs [56,57]. Many reports that highlighted the benefit of intravenous immunoglobulin included patients with early treatment [58]. The benefit for patients with well-established and chronic disease is uncertain [59].

Most recommendations include the combination of glucocorticoids and intravenous immunoglobulin as the first-line treatment [58]. EULAR’s guidelines suggest methylprednisolone as a second-line option, after immunoglobulin, and cyclophosphamide as rescue therapy [6]. The use of other immunosuppressants is less well-documented. Some authors suggest mycophenolate mofetil [58,59] and rituximab [60] as viable options, based on small case series.

We suggest the combination of high-dose glucocorticoids (or intravenous methylprednisolone) combined with intravenous immunoglobulin for new-onset sensory neuronopathy, followed by mycophenolate as maintenance therapy (Table 1).

### 3.7. Multiple Mononeuritis (MM)

MM is thought to be the archetypal clinical presentation of nerve arteriolar obstruction [5]. The cessation of the nerve’s blood supply results in ischemic dysfunction, corresponding to the main functions of the affected nerve, usually with mixed (motor and sensory) deficits. Because most diseases causing MM are systemic, the pathogenic process most often involves other nerves, resulting in a summative pattern sometimes difficult to differentiate from PN [5,41]. Thus, MM can be defined as the simultaneous (although not always synchronous) impairment of at least two peripheral nerves [41].

The pathogenesis of MM includes damage to small arteries and large arterioles ranging approximately 75–300 μm in diameter [5]. They represent the epineural and perineural vessels of the nerves, usually invaded and destroyed by inflammatory cells and/or immune complexes, leading to necrotizing vasculitis and lumen occlusion [61,62].

The disease onset is acute for most patients, but a more prolonged course (more than 2 months of evolution) has been reported in up to 25% of cases [63]. The long nerves of the inferior limbs (e.g., peroneal, sural, and tibial nerves) are the most affected [41,63], but the arms and even cranial nerves are also well described [63]. Because the confluence of nerves can make the patient present with a somewhat symmetric pattern, the assessment for early asymmetry must be pursued [41]. Most cases of MM related to rheumatic conditions represent vasculitic neuropathy; thus, systemic symptoms such as fever, weight loss, and fatigue are frequent. Moreover, one should always seek other organs’ involvement, as it could be the case with hematuria, respiratory distress, and abdominal pain [5].

Considering specifically the neuropathy, the diagnosis is confirmed by NCS, electromyography (EMG), and nerve (and muscle) biopsy [63]. NCS/EMG usually shows an asymmetric axonal loss pattern (sensory and motor action potential’s amplitude reduction) [64]. The electrophysiological studies are important not only for suggesting the diagnosis but also to guide the biopsy because affected nerves probably yield a higher diagnostic accuracy [62]. Despite this, the involvement of nerves is patchy and up to 50% of the findings are not unequivocally diagnostic, supporting the contemporary concept that obtaining a concomitant muscle sample is appreciated when feasible [62,65]. Pathological findings include necrotizing vasculitis of epineural arterioles, mediated by neutrophils and/or monocytes [61]. In connective tissue diseases, concomitant infiltration of perineural space by inflammatory cells can be seen [41], and in SLE, complement and immune complex deposition can also be found [62]. When muscle is obtained, one typically sees the same vasculitic process, without myositis or overt muscle cell necrosis [62].

Because MM’s pathogenesis is so intrinsically linked to vasculitic phenomena, systemic vasculitides are the main diagnosis to pursue [41]. Considering that the vessels involved are those of the epineurium and perineurium, MM is usually associated with small to medium vessel vasculitides. Polyarteritis nodosa (PAN) is an uncommon disease, but it is remarkably associated with MM [37]; so we suggest always considering it in the differentials. Some entities classified by the Chapel Hill Consensus [66] as small vessel vasculitides can also have MM as a clinical manifestation, especially eosinophilic granulomatosis with polyangiitis (EGPA) and cryoglobulinemic vasculitis (CV) [5]. To a lesser extent, the other AAVs should be considered. Systemic autoimmune connective tissue diseases are more related to nerve microvasculitis [5] and most often will produce polyneuropathies [1], as discussed above. Nevertheless, MM in the context of systemic connective tissue diseases is well described and should be remembered. One exception, though, is rheumatoid vasculitis (RV). This entity is rare nowadays with the available pharmacological arsenal for RA because it is related to longstanding and poorly controlled disease [44]. The clinical picture is that of systemic symptoms (especially weight loss), skin ulcers, and MM, in the context of highly active seropositive RA [1,44]. MM can occur in up to 50% of cases [4], so, despite RV being an unusual manifestation of RA, one must remember that RA is by far the most common autoimmune rheumatic condition, deserving an appropriate remark.

**Figure 4:**
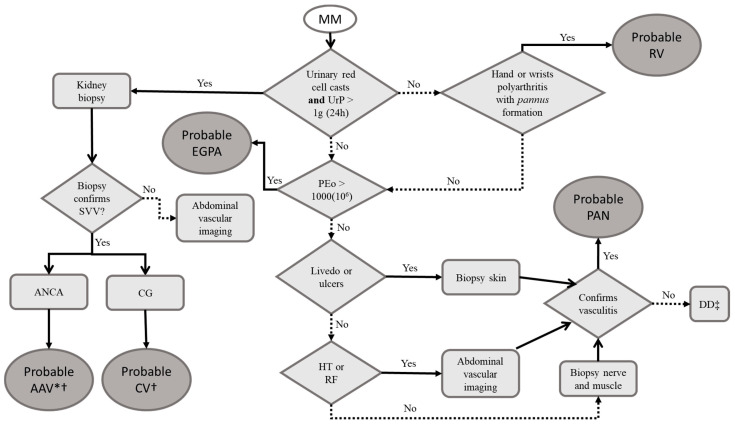
Proposed diagnostic flow for confirmed multiple mononeuropathy. Legend: AAV: antineutrophil cytoplasmic antibody-associated vasculitis; ANCA: antineutrophil cytoplasmic antibody; CG: serum cryoglobulin; CV: cryoglobulinemic vasculitis; DD: differential diagnosis; EGPA: eosinophilic granulomatosis with polyangiitis; HT: hypertension; MM: multiple mononeuropathy; PAN: polyarteritis nodosa; PEo: peripheral eosinophils; RF: renal failure; RV: rheumatoid vasculitis; SVV: small vessel vasculitis; UrP: urinary protein. * If immunofluorescence for antineutrophil cytoplasmic antibodies is perinuclear, confirm with anti-myeloperoxidase; if it is cytoplasmic, confirm with anti-proteinase 3. † If antineutrophil cytoplasmic antibodies are negative, dose cryoglobulins. If cryoglobulins are absent, order antineutrophil cytoplasmic antibodies. If both are negative, treat as antineutrophil cytoplasmic antibody-associated vasculitis (see text) because of the severity. ‡ Consider guiding biopsy by nerve conduction studies and electromyography, or alternatives to multiple mononeuritis, such as hereditary neuropathy with liability to pressure palsies.

The suggested approach for the etiologic workup is summarized in Figure 4.

### 3.8. Treatment of MM

Since MM is a disabling entity, the need for timely intervention to be provided is fairly consensual between guidelines and reviews, regardless of the underlying disease. High-dose glucocorticoids or intravenous glucocorticoids should be prescribed to all patients [67,68,69], except for those with hepatitis-B-related vasculitis, for which antiviral therapy should be the cornerstone of the scheme and immunosuppression should be individualized [70].

For AAV induction of remission, EULAR and the American College of Rheumatology (ACR) guidelines suggest the association of a non-glucocorticoid immunosuppressor to the glucocorticoid, usually cyclophosphamide or rituximab, with a similar level of evidence [46,67]. For PAN and RV, evidence for rituximab is very scarce, so most reviews (based on a limited number of patients) suggest only cyclophosphamide [66,69]. The evidence from CV is also very incipient, but EULAR’s guideline suggests, based on specialists’ opinion, that the notions from AAV could be applied to CV, highlighting that hepatitis-C-related CV should also be treated with antivirals [68].

The remission induction phase usually lasts 3–6 months and the patient should be considered for a remission maintenance phase with methotrexate or azathioprine [67,68]. Patients induced with rituximab with good tolerability can be considered for the maintenance phase with rituximab [67]. The duration of the maintenance phase is uncertain for all the vasculitides described, but more than 18 months is suggested by the ACR for AAV and can be a point of reference for the other conditions [66] (Table 1).

### 3.9. Isolated Mononeuropathies

Mononeuropathies directly related to immune mechanisms in rheumatic diseases are uncommon because these diseases are typically systemic and other nerves or terminations will most likely be concomitantly affected. Nevertheless, isolated cranial neuropathies, especially trigeminal neuropathy, are well described in SS, mixed connective tissue disease, and systemic sclerosis [1,41].

Trigeminal neuropathy is more often that of a purely sensory nature, with numbness and paresthesia in the specific territory of the trigeminal branches (i.e., ophthalmic, maxillary, and mandibular branches). Neuropathic pain may occur, but masticatory muscle involvement is hardly ever present [71].

The diagnosis is suspected by clinical exam, showing hemifacial sensory loss (e.g., in pinprick test) [71]. When motor function is affected, there may be atrophy of masticatory muscles, marks of tongue bites, and deviation of the jaw during speech [71]. NCS/EMG can be used for confirmation, if necessary [72]. MRI of the trigeminal nerve location and related structures is warranted by some authors [71] because compressive neuropathies are more common than immune-mediated ones (when isolated nerves are considered) and the treatment is different.

We suggest that when facing a trigeminal neuropathy without other clinical symptoms (and without tumors documented by imaging), the clinician should consider SS because it can precede the other cardinal features [8].

The treatment of pure sensory trigeminal neuropathy is symptomatic [1]. For neuropathic pain, one should consider duloxetine, pregabalin, or carbamazepine [71].

Many entrapment syndromes can occur in the rheumatic patient because the musculoskeletal tissue represents one of the main sites for inflammation in these diseases and many nerves divide space with soft tissue. Therefore, the inflammation of these constricted areas are easily predisposed to neuropathy. Some remarkable diseases include carpal tunnel syndrome (CTS) and tarsal tunnel syndrome (TTS). They are reviewed briefly because the intent of the review is to focus on immune-mediated conditions.

In CTS, patients might complain initially of tingling, numbness, or pain in the hand, sometimes irradiating to the shoulder, and worse at the end of the night or morning, right after waking up. The symptoms can also occur after repetitive activities involving wrist flexion. With the progression of the disease, symptoms can occur outside of the morning window and muscular dysfunction may appear, with grip clumsiness or even overt weakness and thenar hypotrophy [73]. The disease is related to entrapment of the median nerve inside the carpal tunnel, so entities that promote extensive synovitis of the wrists are good candidates for etiologic agents. Nevertheless, CTS is a very common condition and no primary inflammatory disease is present in most cases [74].

TTS represents the entrapment of the posterior tibial nerve within the tarsal tunnel, formed by the flexor’s retinaculum and the tarsal bones [75]. Most patients present at first with pain, numbness, or a tingling sensation behind the medial malleolus, medial portion of the foot, heel, and plantar surface. Because of the venous drainage, symptoms are typically worse during daily activities, especially when the patient stands for extended periods [75], but as with any neuropathic pain, symptoms can also occur in the night. In advanced disease, motor impairment and interdigital atrophy can be seen. TTS is associated with diabetes, hind foot intrinsic deformities, trauma, and scarring, among others. Only a small portion of patients have inflammatory synovitis as a background, but it is a well-described cause [75].

Among the rheumatic autoimmune diseases related to CTS and TTS, RA is the most probable because it is more related to intense inflammatory synovitis. Peripheral spondyloarthritis is also remarkable, but it is not within the scope of this review, which is focused more on immune-mediated neuropathies.

Regarding the treatment of the entrapment neuropathies related to synovial inflammation, efforts should be made to control the underlying disease itself [44]. Considering RA, methotrexate will most likely be the first option, associated with anti-tumoral necrosis factor (anti-TNF) biologics in the case of failure [76]. Injections of glucocorticoids can promote relief until the immunosuppressors are adjusted [44].

## 4. Conclusions

Because the rheumatic-disease-related peripheral neuropathies can occur with a vast myriad of signs and symptoms, diagnosis is often hard and the narrow therapeutic window overlooked. As physical impairment is already a challenge in these patients because of advanced age and musculoskeletal manifestations, early and aggressive treatment of neurological manifestations is imperative. Future developments involving genetic and antibody profiles might help to predict peripheral neuropathies, but, at present, the knowledge is still scarce on the subject. Thus, we believe that a stepwise approach for diagnosis such as the one presented can facilitate the clinical reasoning until future tools are refined.

## Figures and Tables

**Table 1:** Suggested schemes for induction and maintenance of remission of the discussed autoimmune rheumatic-disease-related neuropathies.

Condition	Induction	Maintenance
Small fiber neuropathy	Intravenous methylprednisolone pulse; intravenous immunoglobulin *	Evidence limited, consider gabapentin and/or tramadol for neuropathic pain
Axonal sensory neuropathy/sensorimotor polyneuropathy	Intravenous methylprednisolone pulse, intravenous cyclophosphamide pulses	Azathioprine, methotrexate, mycophenolate mofetil, rituximab
Sensory neuronopathy	Intravenous methylprednisolone pulse; intravenous immunoglobulin *	Mycophenolate mofetil
Multiple mononeuritis	Intravenous methylprednisolone pulse, intravenous cyclophosphamide pulses, rituximab	Azathioprine, methotrexate, mycophenolate mofetil, rituximab

Suggested doses: intravenous methylprednisolone: 1 g/day for 3 days; intravenous immunoglobulin: 1 g/kg/day for 2 days; intravenous cyclophosphamide pulses: 500 mg/day every 15 days for 6 doses; oral methotrexate: 10–25 mg/week; oral azathioprine: 2–3 mg/kg/day; oral mycophenolate mofetil: 2–3 g/day; rituximab: 1 g on days 1 and 15, followed by 500 mg every 6 months. * Intravenous immunoglobulin therapy refers to the infusion of a mixture of polyvalent IgG from thousands of donors. There is no sufficient evidence favoring a particular brand of human immunoglobulin.

## Data Availability

Not applicable.
